# The N-terminal region of serum amyloid A3 protein activates NF-κB and up-regulates MUC2 mucin mRNA expression in mouse colonic epithelial cells

**DOI:** 10.1371/journal.pone.0181796

**Published:** 2017-07-24

**Authors:** Manami Tashiro, Ami Iwata, Marika Yamauchi, Kaori Shimizu, Ayaka Okada, Naotaka Ishiguro, Yasuo Inoshima

**Affiliations:** 1 Laboratory of Food and Environmental Hygiene, Cooperative Department of Veterinary Medicine, Faculty of Applied Biological Sciences, Gifu University, Gifu, Japan; 2 The United Graduate School of Veterinary Sciences, Gifu University, Gifu, Japan; 3 Education and Research Center for Food Animal Health, Gifu University (GeFAH), Yanagido, Gifu, Gifu, Japan; Memorial University of Newfoundland, CANADA

## Abstract

Serum amyloid A (SAA) is the major acute-phase protein and a precursor of amyloid A (AA) in AA amyloidosis in humans and animals. SAA isoforms have been identified in a wide variety of animals, such as SAA1, SAA2, SAA3, and SAA4 in mouse. Although the biological functions of SAA isoforms are not completely understood, recent studies have suggested that SAA3 plays a role in host defense. Expression of SAA3 is increased on the mouse colon surface in the presence of microbiota *in vivo*, and it increases mRNA expression of mucin 2 (MUC2) in murine colonic epithelial cells *in vitro*, which constitutes a protective mucus barrier in the intestinal tract. In this study, to identify responsible regions in SAA3 for MUC2 expression, recombinant murine SAA1 (rSAA1), rSAA3, and rSAA1/3, a chimera protein constructed with mature SAA1 (amino acids 1–36) and SAA3 (amino acids 37–103), and vice versa for rSAA3/1, were added to murine colonic epithelial CMT-93 cells, and the mRNA expressions of MUC2 and cytokines were measured. Inhibition assays with NF-κB inhibitor or TLR4/MD2 inhibitor were also performed. Up-regulation of MUC2 mRNA expression was strongly stimulated by rSAA3 and rSAA3/1, but not by rSAA1 or rSAA1/3. Moreover, NF-κB and TLR4/MD2 inhibitors suppressed the increase of MUC2 mRNA expression. These results suggest that the major responsible region for MUC2 expression exists in amino acids 1–36 of SAA3, and that up-regulations of MUC2 expression by SAA3 and SAA3/1 are involved with activation of NF-κB via the TLR4/MD2 complex.

## Introduction

Serum amyloid A (SAA) is the major acute-phase protein in humans, most mammals, and avians [1]. SAA is also known as a precursor protein of amyloid A (AA) in AA amyloidosis, which is a long-term complication of several chronic inflammatory disorders such as rheumatoid arthritis and juvenile inflammatory arthritis [2]. Differences in amino acid sequence have indicated the existence of multiple SAA isoforms, such as SAA1, 2, 3, and 4 in mouse [1]. SAA1 and SAA2 are well known as main acute-phase isoforms, which are mainly expressed in the liver [1]. SAA3, which is up-regulated during acute and chronic inflammatory responses, is predominantly expressed by macrophages and other cells, including adipocytes, epithelial cells, and endothelial cells in mice [3–5]. A fourth isoform, SAA4, is constitutively expressed in the liver [6]. In addition to the difference in primary synthesis site, SAA3 is unique among SAA family members. Among the four SAA isoforms, SAA1, 2, and 4, but not SAA3, have been shown to be associated with high density lipoprotein in mice [7]. Moreover, SAA1 (GenBank accession no. BC087933) and SAA2 (M11130) genes share 95.1% and 92.6% sequence identities in 369 nucleotides and 122 amino acids, respectively, whereas respective identities between SAA1 and SAA3 (NM011315) are 74.3% and 64.7%.

Although the biological functions of SAA isoforms are not completely understood, recent studies have suggested that SAA may play a role in host defense. Shah et al. [8] reported that SAA1 binds to outer membrane protein A of *Escherichia coli* and *Pseudomonas aeruginosa* for opsonization, and suggested that SAAs play a role in innate immunity by opsonization of gram-negative bacteria. However, the expression of SAA3, but not SAA1 or 2, is increased on the mouse colon surface in the presence of microbiota [5], and lipopolysaccharide (LPS) strongly induces mRNA expression of SAA3 in murine colonic epithelial CMT-93 cells [5, 9, 10]. Moreover, our previous study demonstrated that SAA3, but not SAA1, increases mRNA expression of mucin 2 (MUC2) in CMT-93 cells [10]. MUC2 is a high molecular weight gel-forming glycoprotein that is secreted into the gut lumen and forms the major mucin component of the protective mucus barrier in the intestinal tract [11]. These results suggest that SAA3 stimulated by LPS relates to intestinal immunity. However, the differences between SAA3 and other SAAs are not fully understood. The mechanism for the induction of MUC2 expression by SAA3 also remains unclear.

In this study, to identify the responsible amino acid sequence region of SAA3 for MUC2 expression, recombinant murine SAA1 (rSAA1), rSAA3, and rSAA1/3, a chimera protein constructed with mature SAA1 (amino acids 1–36) and SAA3 (amino acids 37–103), and vice versa for rSAA3/1, were added to murine colonic epithelial CMT-93 cells, and the mRNA expressions of MUC2 and cytokines were analyzed. Moreover, inhibition assays using NF-κB inhibitor and toll-like receptor 4 (TLR4)/MD2 inhibitor were performed. We demonstrated that MUC2 mRNA expression was significantly up-regulated by rSAA3 and rSAA3/1 compared with rSAA1 and rSAA1/3. In addition, both NF-κB inhibitor and TLR4/MD2 inhibitor suppressed MUC2 mRNA expression by rSAA3 and rSAA3/1, respectively. These results suggest that the major responsible region for MUC2 expression exists in amino acids 1–36 of SAA3, and that up-regulation of MUC2 expression by SAA3 is involved with the activation of NF-κB via the TLR4/MD2 complex.

## Materials and methods

### Cells

The murine large intestinal epithelial cell line, CMT-93 (CCL-223), was purchased from the European Collection of Authenticated Cell Cultures (ECACC) and maintained in Dulbecco’s modified Eagle’s minimal essential medium (DMEM, Wako, Osaka, Japan) containing 100 U/ml penicillin, 100 μg/ml streptomycin (Gibco, Grand Island, NY), and 10% fetal bovine serum (PAA Laboratories GmbH, Pasching, Austria).

### rSAAs

**Fig 1:**
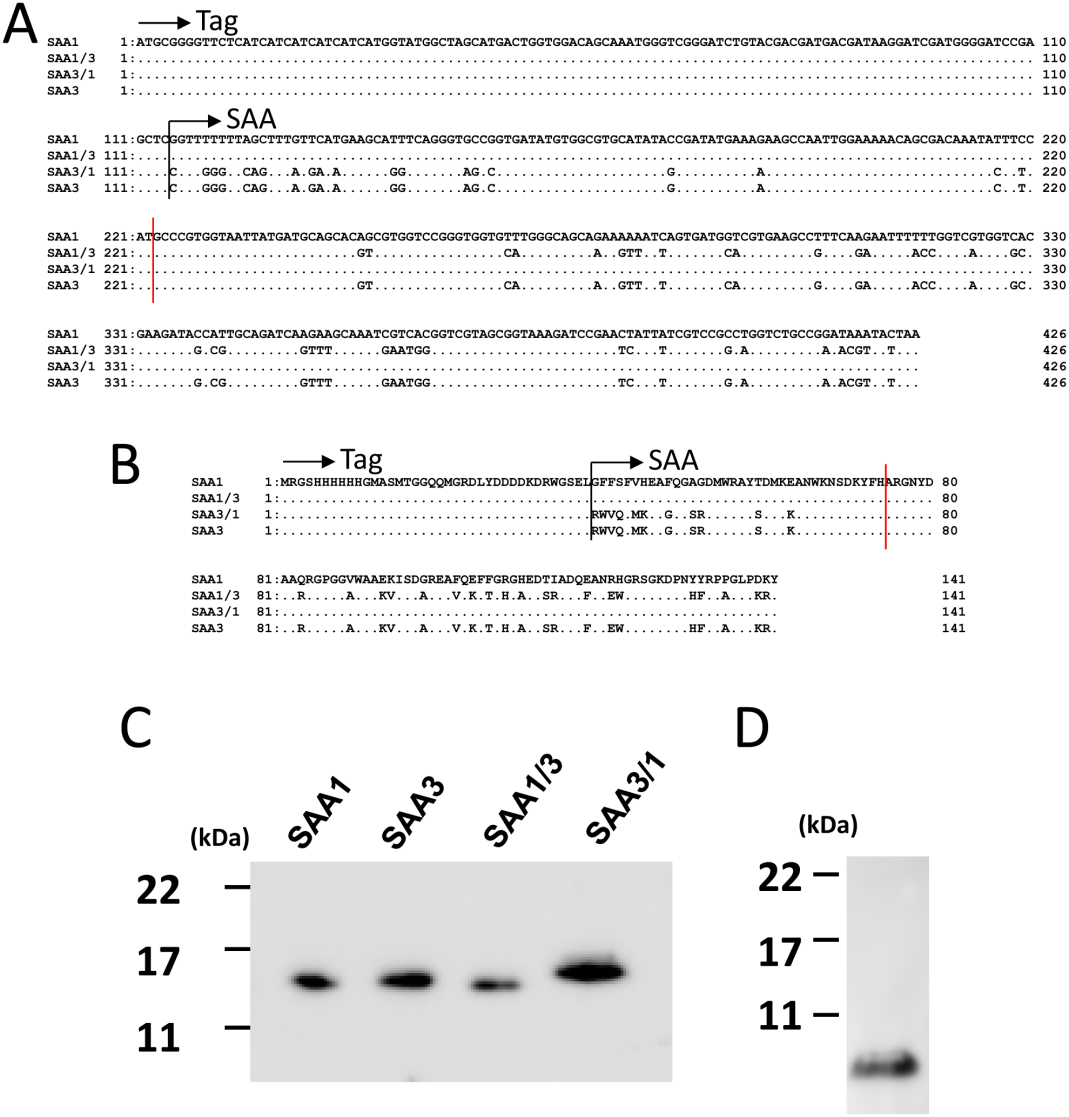
Production of rSAA1, rSAA3, rSAA1/3, and rSAA3/1 proteins. **(A)** Alignment of nucleotide sequences of rSAAs optimized for *E*. *coli* expression combined with tag sequence from the pRSET A vector. Consensus nucleotides are shown by dots. Red vertical line indicates the switching point of sequence for chimera proteins SAA1/3 and SAA3/1. **(B)** Alignment of amino acid sequences of rSAAs, identical to those of mouse SAA1 (BC087933) and SAA3 (NM011315). Consensus amino acids are shown by dots. Red vertical line indicates the switching point of sequence for chimera proteins SAA1/3 and SAA3/1. **(C)** Western blotting analysis of expressed rSAAs using an anti-Xpress monoclonal antibody (R910-25, Invitrogen). As expected (http://web.expasy.org/compute_pi/), the molecular weights of expressed rSAA1, rSAA3, rSAA1/3, and rSAA3/1 were approximately 16.1, 16.1, 16.0, and 16.3 kDa, respectively. **(D)** WB analysis of expressed tag protein of the pRSET A vector using an anti-Xpress monoclonal antibody.

Recombinant murine SAA1, rSAA3, rSAA1/3, and rSAA3/1 were constructed as follows. Nucleotide sequences excluding the signal sequence (nucleotides 1–57 [10]) of murine SAA1 (BC087933) and SAA3 (NM011315) were optimized for an *E*. *coli* protein expression system without changing the amino acid sequence by Invitrogen (Carlsbad, CA). As for rSAA1/3, the nucleotide sequence of optimized SAA1 (nucleotides 1–108; amino acids 1–36) was combined with SAA3 (nucleotides 109–312; amino acids 37–103), and vice versa for rSAA3/1 (Fig 1A and 1B). A synthesized DNA fragment flanked with *Sac* I and *Kpn* I sites was digested with *Sac* I and *Kpn* I (Toyobo, Osaka, Japan) and cloned between the *Sac* I and *Kpn* I sites of the pRSET A expression vector (Invitrogen).

### Expression and purification of rSAA

After confirmation of their sequences, the plasmids were transformed into *E*. *coli* BL21 (DE3) pLysS (Invitrogen). Cultured *E*. *coli* in Magic Media (Invitrogen) was collected and rSAAs were extracted and purified as described in detail previously [10]. Tag protein from the pRSET A vector was also expressed and purified. Coomassie brilliant blue (CBB) staining and Western blotting (WB) analysis were performed as described previously [10]. Peroxidase activity in WB was visualized by an LAS 4000mini (Fujifilm, Tokyo, Japan).

### Quantitative real-time PCR

CMT-93 cells were seeded at 4–6×10^5^ cells in 6-well plates and incubated for 15±1 h before experiment. CMT-93 cells were treated with rSAAs at 37°C for 2 h, washed with PBS, and total RNA was extracted immediately using an RNeasy Mini kit (Qiagen, Hilden, Germany) following the manufacturer’s instructions. As for inhibitor assays, tumor necrosis factor (TNF)-α inhibitor (Enzo Life Sciences, Lausen, Switzerland); NF-κB inhibitor, CAPE (Calbiochem, EMD Chemicals, San Diego, CA); or TLR4/MD2 inhibitor, TAK-242 (MedChem Express, Monmouth Junction, NJ) was added to cells at 37°C for 1 h before incubation with rSAAs. Isolated RNA was quantified using a spectrophotometer GeneQuant 100 (GE Healthcare) and stored at -80°C until use. Contaminating DNA was removed with DNase I (Invitrogen), and cDNA was synthesized using the SuperScript III First-Strand Synthesis System SuperMix for qRT-PCR (Invitrogen) according to the manufacturer’s instructions. Quantitative real-time PCR was performed using a Fast SYBR Green PCR Master Mix (Applied Biosystems, Foster City, CA) [10]. To investigate mRNA expressions of mucin 2 (MUC2), TNF-α, interleukin (IL)-6, inhibitor κB (IκB)-α, and glyceraldehyde-3-phosphate dehydrogenase (GAPDH), their specific primers [12–17] were used for real-time PCR (S1 Table). Regenerating islet-derived 3 (REG III)-γ, α-defensin (Def), β-Def-3, and β-Def-4 are anti-bacterial proteins secreted by intestinal epithelial cells by sensing bacteria and bacterial antigens as well as mucins, and contribute to the innate immunity of the intestine [18, 19]. Therefore, mRNAs of REG III-γ, α-Def, β-Def-3, and β-Def-4 were also examined by quantitative real-time PCR. Results were normalized to the expression of GAPDH mRNA as an endogenous gene and fold-change relative to control levels were determined by the ΔΔC_t_ method [20]. For verification of specific amplification, a melting-curve analysis of amplification products was performed at the end of each PCR reaction. All experiments were replicated at least three times.

### Measurement of cytokines in cell culture supernatant

CMT-93 cells were seeded at 1.2×10^5^ cells in 24-well plates and incubated for 15±1 h before experiments. After incubation, CMT-93 cells were treated with rSAAs at 37°C for 24 h. The amounts of cytokines in cell culture supernatants were measured using a BD Cytometric Bead Array (CBA) Mouse T helper type 1 (Th1)/Th2/Th17 Cytokine Kit [IL-2, IL-4, IL-6, IL-10, IL-17A, TNF-α, and interferon (IFN)-γ] (BD Biosciences, Franklin Lakes, NJ), according to the manufacturer’s instructions. Data were acquired on a flow cytometer FACSCantoII (BD Biosciences) and analyzed with FACSDiva software (BD Biosciences).

### Statistical analyses

The data were collected from at least three independent experiments, expressed as means ± SD, and analyzed for statistical significance by unpaired *t*-tests.

## Results

### Expression of rSAA1, rSAA3, rSAA1/3, and rSAA3/1

rSAA1, rSAA3, rSAA1/3, and rSAA3/1 proteins were generated. Amino acid sequence identity between rSAA1 and rSAA3, rSAA3 and rSAA1/3, and rSAA3 and rSAA3/1 were approximately 65%, 89%, and 81%, respectively. Expressed rSAAs were confirmed by CBB staining (data not shown) and WB (Fig 1C). As expected (http://web.expasy.org/compute_pi/), the molecular weights of expressed rSAA1, rSAA3, rSAA1/3, and rSAA3/1 were approximately 16.1, 16.1, 16.0, and 16.3 kDa, respectively.

### Induction of MUC2 mRNA in CMT-93 cells by rSAAs

**Fig 2:**
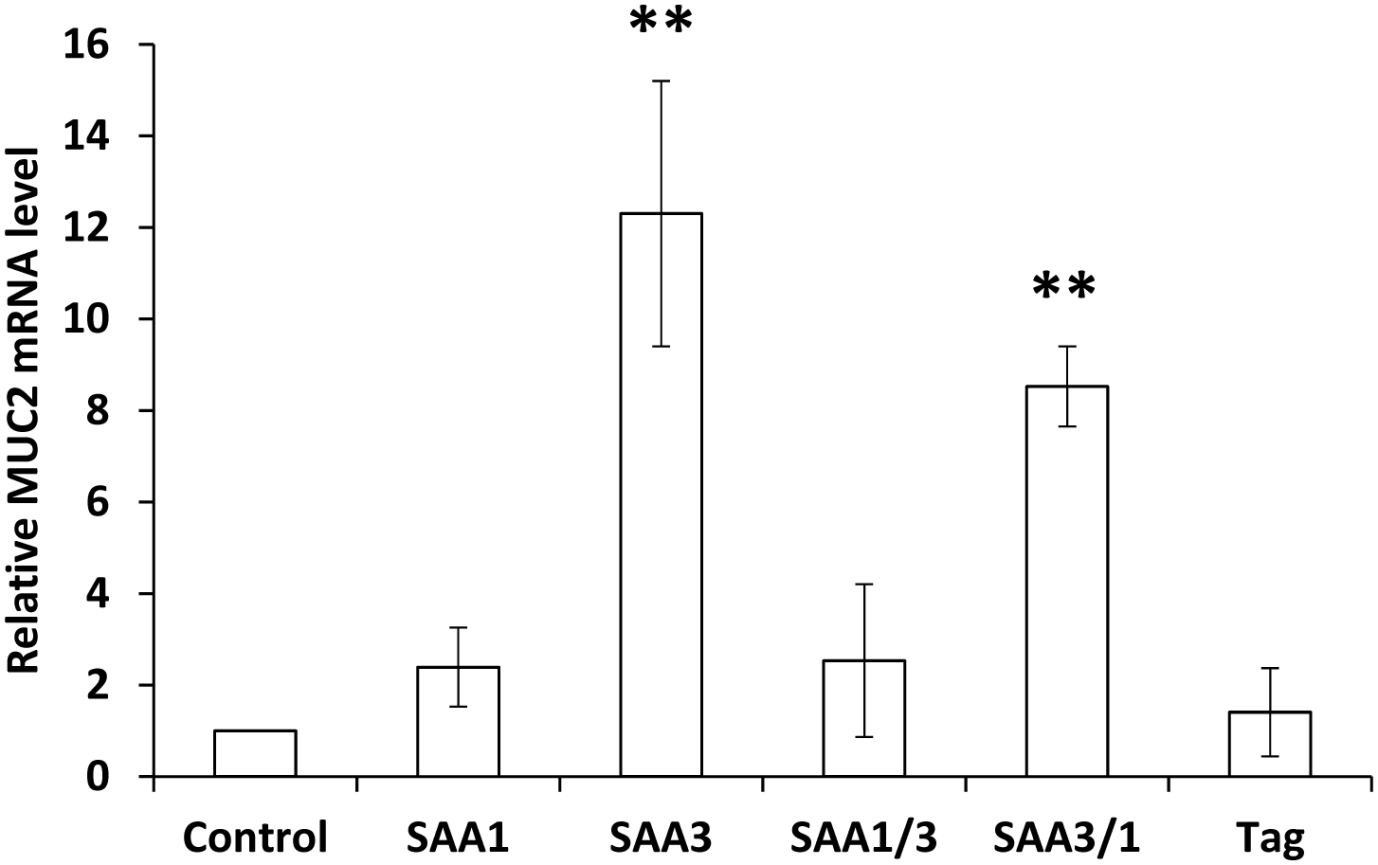
Induction of MUC2 mRNA expressions in CMT-93 cells by rSAAs. CMT-93 cells were incubated with rSAAs or tag protein (100 μg/ml) for 2 h at 37°C. The relative expression levels of MUC2 mRNA were corrected with GAPDH and then compared with control. Data are the means of six independent observations (except for tag protein, which were the means of three independent observations) with the standard deviations represented by vertical bars. Asterisk indicates significant difference compared with the control. **p<0.01.

To identify the responsible region in SAA3 for MUC2 expression, CMT-93 cells were incubated with rSAA1, rSAA3, rSAA1/3, or rSAA3/1. MUC2 mRNA expression in CMT-93 cells was strongly induced by rSAA3 and rSAA3/1, but not by rSAA1, rSAA1/3, and tag proteins (Fig 2). These results suggested that the responsible region for MUC2 expression exists in amino acids 1–36 of SAA3. rSAAs did not affect the mRNA expressions of REG III-γ, α-Def, β-Def-3, or β-Def-4 (data not shown).

### Induction of cytokine mRNA and protein expressions by rSAAs

**Fig 3:**
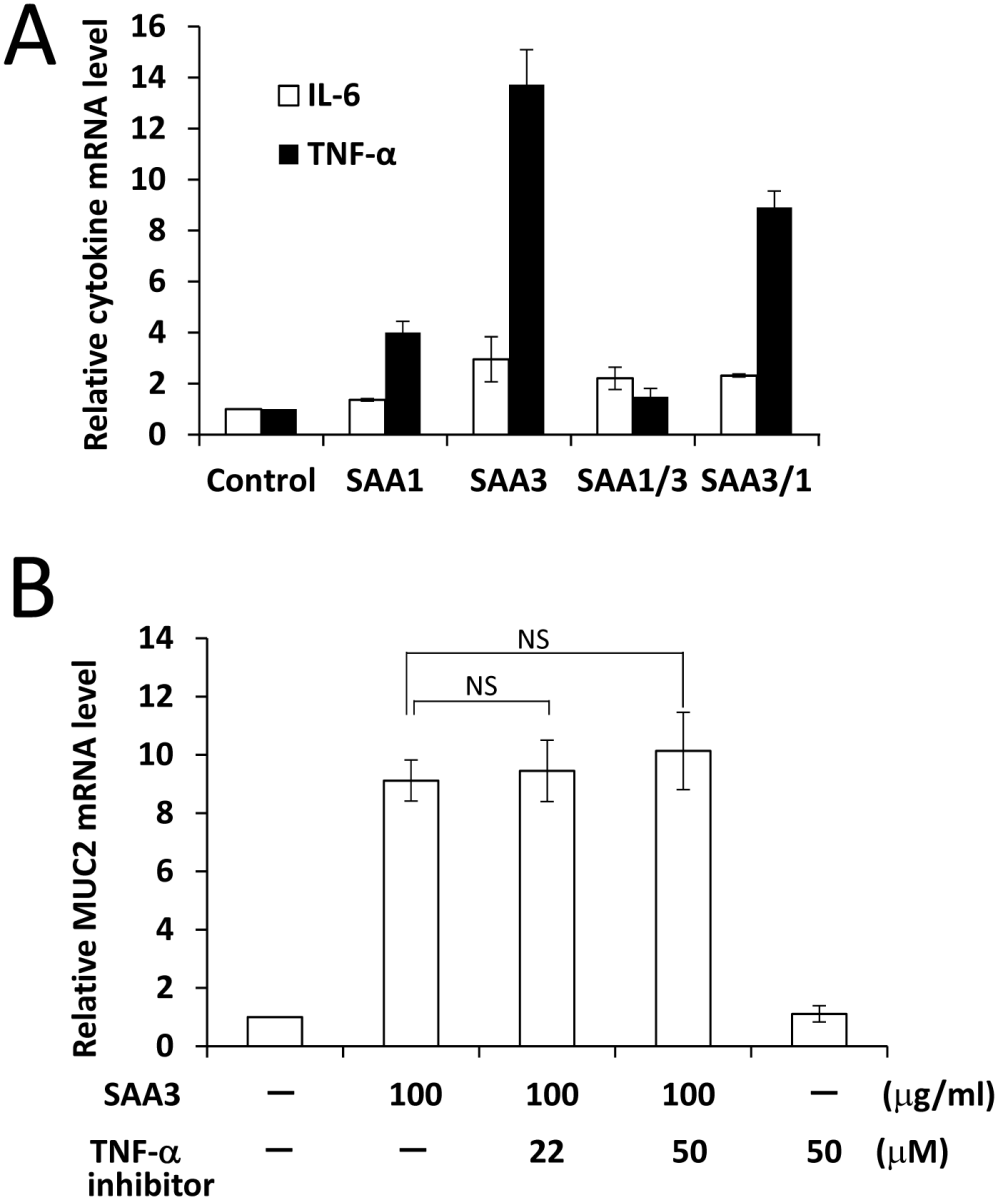
Induction of cytokine mRNAs and protein expressions in CMT-93 cells by rSAAs. **(A)** CMT-93 cells were incubated with rSAAs (100 μg/ml) for 2 h at 37°C. The relative expression levels of TNF-α and IL-6 mRNA were corrected with GAPDH and then compared with the control. Data are the means of four independent observations with the standard deviations represented by vertical bars. **(B)** Effect of TNF-α inhibitor on induction of MUC2 mRNA expression by rSAAs. CMT-93 cells were incubated with TNF-α inhibitor before adding rSAAs. The relative expression levels of MUC2 mRNA were corrected with GAPDH and then compared with the control. Data are the means of four independent observations with the standard deviations represented by vertical bars. NS, no significant difference.

Our previous study showed that both SAA1 and SAA3 enhanced IL-6 and TNF-α mRNA expression [10]. To confirm that rSAA1/3 and rSAA3/1 induce cytokines, CMT-93 cells were treated with rSAA1, rSAA3, rSAA1/3, or rSAA3/1, and mRNA and protein expressions of inflammatory cytokines were estimated. There was little difference in IL-6 mRNA expression among the rSAA treatments (Fig 3A). On the other hand, rSAA1, rSAA3, and rSAA3/1 enhanced TNF-α mRNA expression. In particular, rSAA3 and rSAA3/1 intensively induced TNF-α. Although IL-6 and TNF-α proteins were induced by rSAA3 and rSAA3/1, other cytokines were not induced (Table 1). We therefore confirmed that both IL-6 and TNF-α were induced by rSAA3 and rSAA3/1 at the protein level.

**Table 1:** Cytokine profiles in supernatant after incubation with rSAAs (pg/ml±SD).

	Control	SAA1	SAA3	SAA1/3	SAA 3/1
IL-2	ND	ND	ND	ND	ND
IL-4	ND	2.12±3.67	ND	2.40±4.15	2.78±4.81
IL-6	3.46±0.76	8.46±0.64	93.75±2.84	7.11±1.88	76.99±6.02
IL-10	3.89±6.73	3.30±5.71	2.03±3.51	ND	1.27±2.21
IL-17	ND	ND	ND	ND	ND
TNF-α	8.61±0.75	13.25±0.54	262.08±31.56	30.41±21.94	91.38±3.09
IFN-γ	ND	ND	ND	ND	ND

ND, not detectable

Moreover, to examine whether TNF-α induced by rSAA3 affected the induction of MUC2 expression, cells were exposed to TNF-α inhibitor with rSAA3. TNF-α inhibitor did not significantly affect the induction of MUC2 mRNA expression (Fig 3B), meaning that TNF-α might not contribute to the induction of MUC2 mRNA expression at 2 h.

### Induction of IκB-α mRNA expression by rSAAs

**Fig 4:**
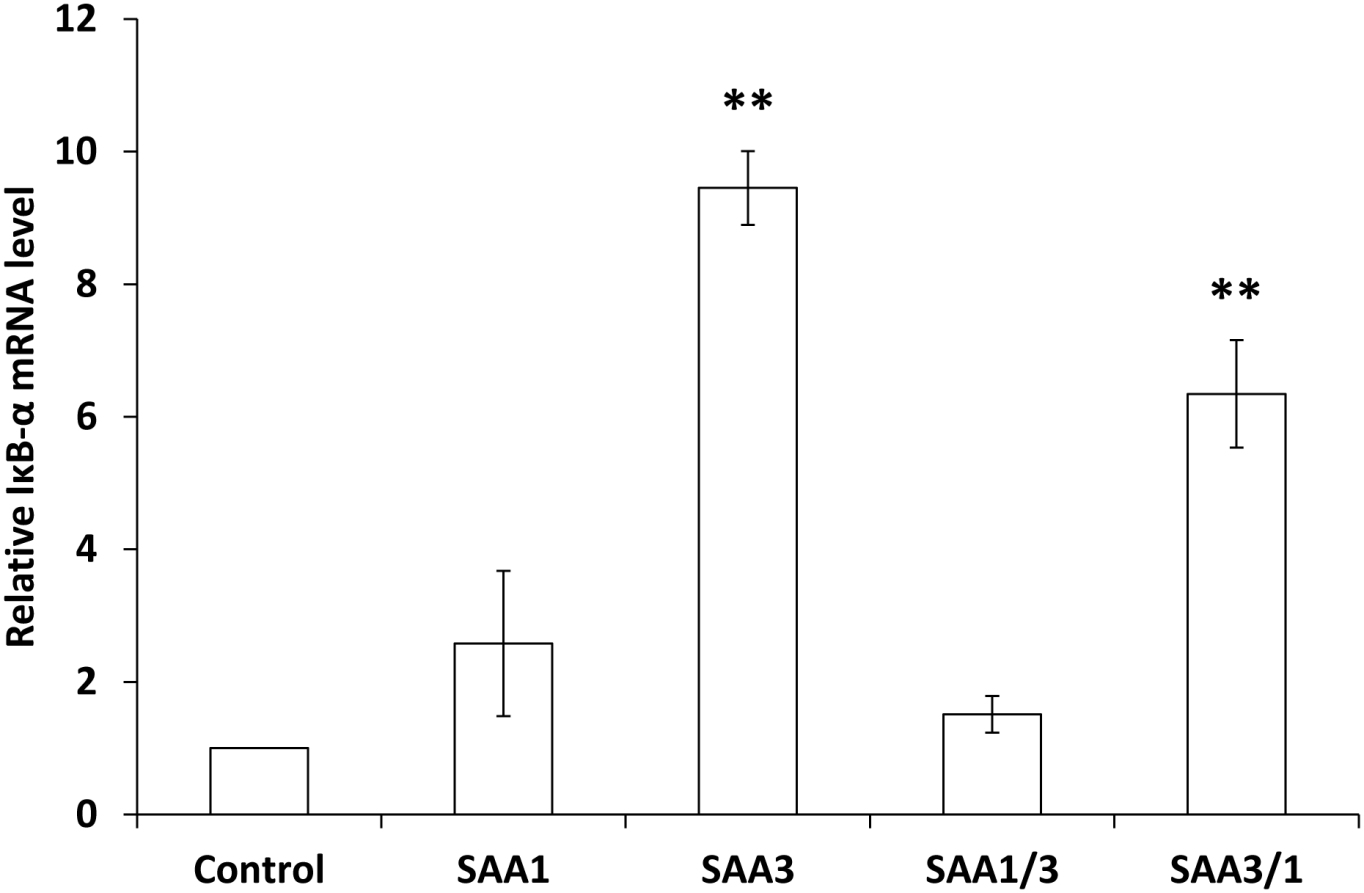
Induction of IκB-α expressions in CMT-93 cells by rSAAs. CMT-93 cells were incubated with rSAAs (100 μg/ml) for 2 h at 37°C. The relative expression levels of IκB-α mRNA were corrected with GAPDH and then compared with the control. Data are the means of four independent observations with the standard deviations represented by vertical bars. Asterisk indicates significant difference compared with the control. **p<0.01.

It has been reported that SAA3 is an endogenous peptide ligand for the TLR4/MD2 complex, the activated NF-κB signaling pathway in metastatic mouse lung [3], and the TLR4/MD2 complex expressed in colonic epithelial cells [5]. Therefore, we considered that SAA proteins may induce MUC2 mRNA expression through the NF-κB signaling pathway. To test whether the NF-κB signaling pathway was activated by SAAs, cells were treated with rSAA1, rSAA3, rSAA1/3, or rSAA3/1, and then IκB-α mRNA expressions were examined, because IκB-α mRNA levels quantitatively result in NF-κB activation [21]. IκB-α mRNA expressions were strongly induced by rSAA3 and rSAA3/1 (Fig 4); these results were consistent with those obtained from MUC2 mRNA expressions (Fig 2).

### NF-κB inhibitor reduced MUC2, TNF-α, and IL-6 mRNA

**Fig 5:**
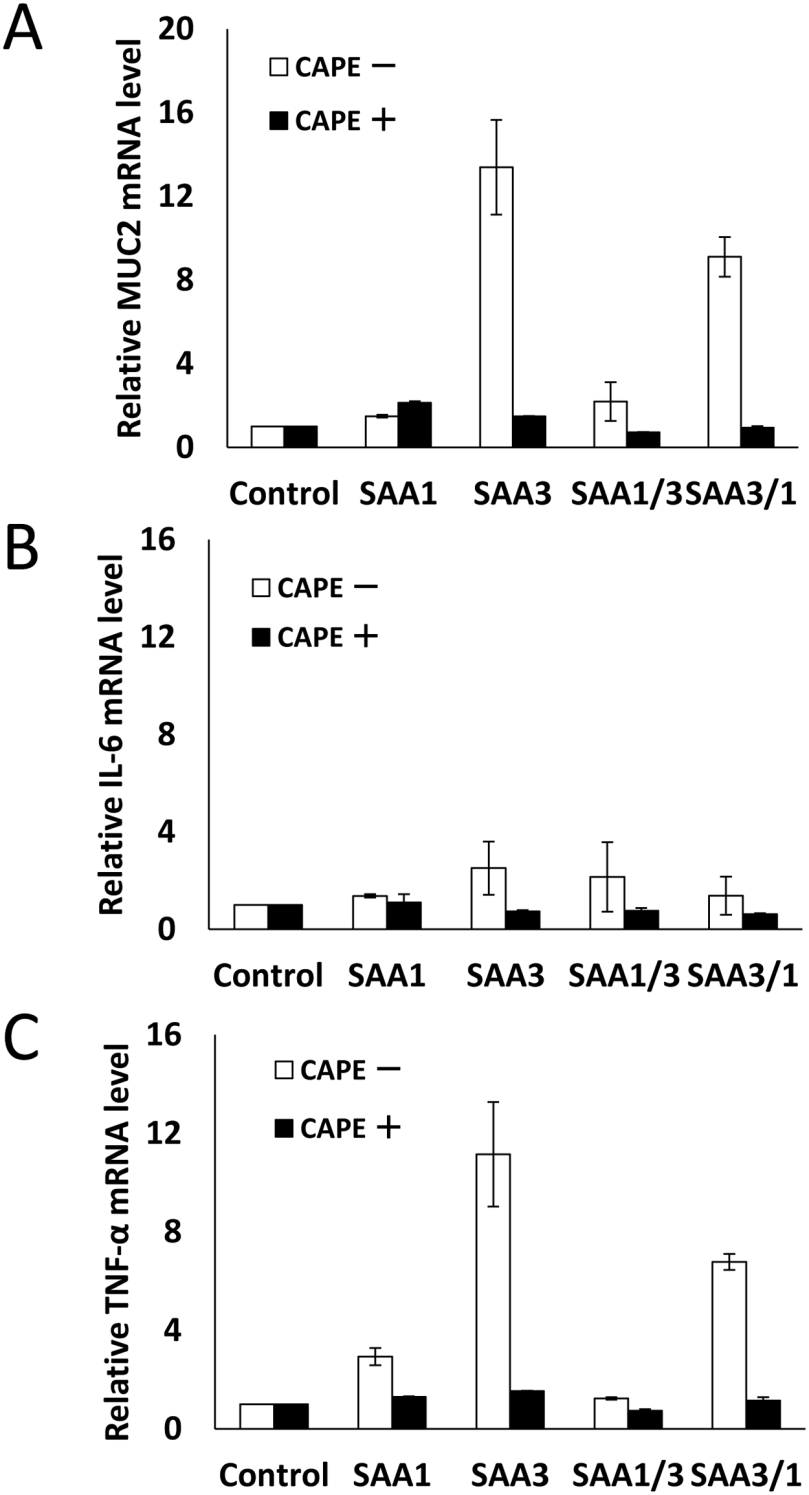
Effect of NF-κB inhibitor on induction of MUC2, IL-6, and TNF-α mRNA expression by rSAAs. CMT-93 cells were incubated with 25μg/ml of NF-κB inhibitor, CAPE, before adding rSAAs. The relative expression levels of (A) MUC2, (B) IL-6, and (C) TNF-α mRNA were corrected with GAPDH and then compared with the control. Data are the means of four independent observations with standard deviations represented by vertical bars.

To test whether SAA proteins regulate MUC2 expression through the NF-κB signaling pathway, cells were exposed to an NF-κB inhibitor, CAPE, before incubation with rSAAs. The NF-κB inhibitor reduced MUC2 mRNA expression by rSAAs (Fig 5). Similarly, the NF-κB inhibitor reduced IL-6 and TNF-α mRNA expressions by rSAAs. These results suggest that rSAA proteins can activate the NF-κB signaling pathway to up-regulate the expressions of MUC2, TNF-α and IL-6 mRNA.

### TLR4/MD2 inhibitor reduced MUC2, IL-6, TNF-α, and IκB-α mRNA expressions by rSAAs

**Fig 6:**
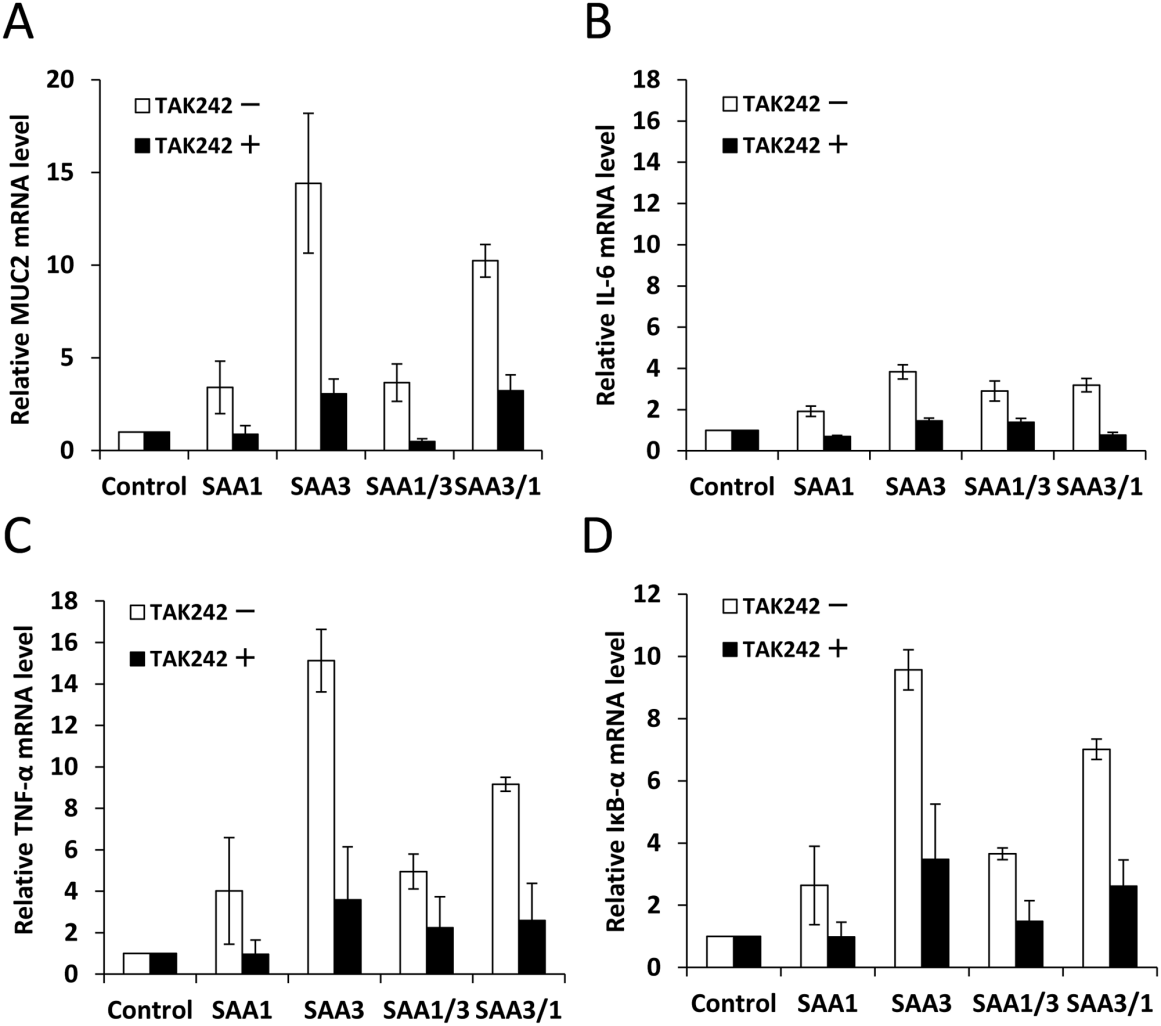
Effect of TLR4/MD2 inhibitor on induction of MUC2, IL-6, TNF-α, and IκB-α mRNA expressions by rSAAs. CMT-93 cells were incubated with 1μM of TLR4/MD2 inhibitor, TAK-242, before adding rSAAs. The relative expression levels of (A) MUC2, (B) IL-6, (C) TNF-α, and (D) IκB-α mRNA were corrected with GAPDH and then compared with the control. Data are the means of four independent observations with the standard deviations represented by vertical bars.

To examine whether SAA proteins were recognized by the TLR4/MD2 complex, and whether MUC2 was induced through the TLR4/MD2 complex, cells were exposed to a TLR4/MD2 inhibitor, TAK-242, before incubation with rSAAs. The TLR4/MD2 inhibitor reduced MUC2, IL-6, and TNF-α mRNA expressions (Fig 6). IκB-α mRNA expressions were suppressed in the presence of the TLR4/MD2 inhibitor, confirming its effect. These results suggest that SAA proteins are associated with the TLR4/MD2 complex in murine colonic epithelial cells and can induce MUC2, IL-6, and TNF-α.

## Discussion

In this study, we produced rSAA1, rSAA3, and the chimeras, rSAA1/3 and rSAA3/1. Intensive up-regulation of MUC2 mRNA expression by rSAA3 and rSAA3/1, compared with rSAA1 and rSAA1/3, indicated that the responsible region for stimulation of MUC2 expression exists in amino acids 1–36 of SAA3. Moreover, both NF-κB and TLR4/MD2 inhibitor suppressed the induction of MUC2 expression by rSAA3 and rSAA3/1, respectively. These results suggest that a region within amino acids 1–36 of SAA3 is associated with the TLR4/MD2 complex and activates NF-κB to induce MUC2 expression. A previous study has reported that synthesized peptides of murine SAA3 (amino acids 24–38) show a marked affinity to the TLR4/MD2 complex, mainly MD2, while other peptides of various SAA3 regions do not [22]. Produced rSAA3 and rSAA3/1 contained the same amino acid sequence (24–36) and may have potent affinity to the TLR4/MD2 complex. SAA3 binds to TLR4/MD2 and activates NF-κB [5, 22]. Therefore, the 1–36 amino acid region of SAA3 likely has the ability to activate the NF-κB signaling pathway via TLR4/MD2 and induce MUC2 expression in CMT-93 cells.

Since it has been reported that TNF-α up-regulates MUC2 expression in human intestinal cancer LS180 cells [23] and colonic epithelial HT-29 cells [24], it is possible that the induction of MUC2 mRNA expression observed in this study is not an effect of SAA3, but is rather due to the effect of TNF-α induced by rSAA3 because TNF-α also enhances NF-κB independently of the TLR4/MD2-NF-κB signaling pathway. However, inhibition assays revealed that a TNF-α inhibitor did not affect the induction of MUC2 mRNA expression, suggesting that TNF-α is not necessary for MUC2 mRNA expression by SAA3. In addition to the up-regulation of MUC2 expression by SAA3 and TNF-α, it has been reported that TNF-α induces SAA3 mRNA expression in CMT-93 cells [5] and mouse granulosa tumor OV3121-1 cells [25]. Moreover, IL-6 induces other mucins, MUC4 and MUC5B, aside from MUC2 [23, 26], and also induces SAA3 expression [27–29]. These results suggest that MUC2 and other mucins are consecutively produced in cooperation with SAA3 and cytokines, such as TNF-α and IL-6, and that SAA3 plays a role in intestinal immunity with cytokines to protect epithelial cells from bacterial infection.

**Fig 7:**
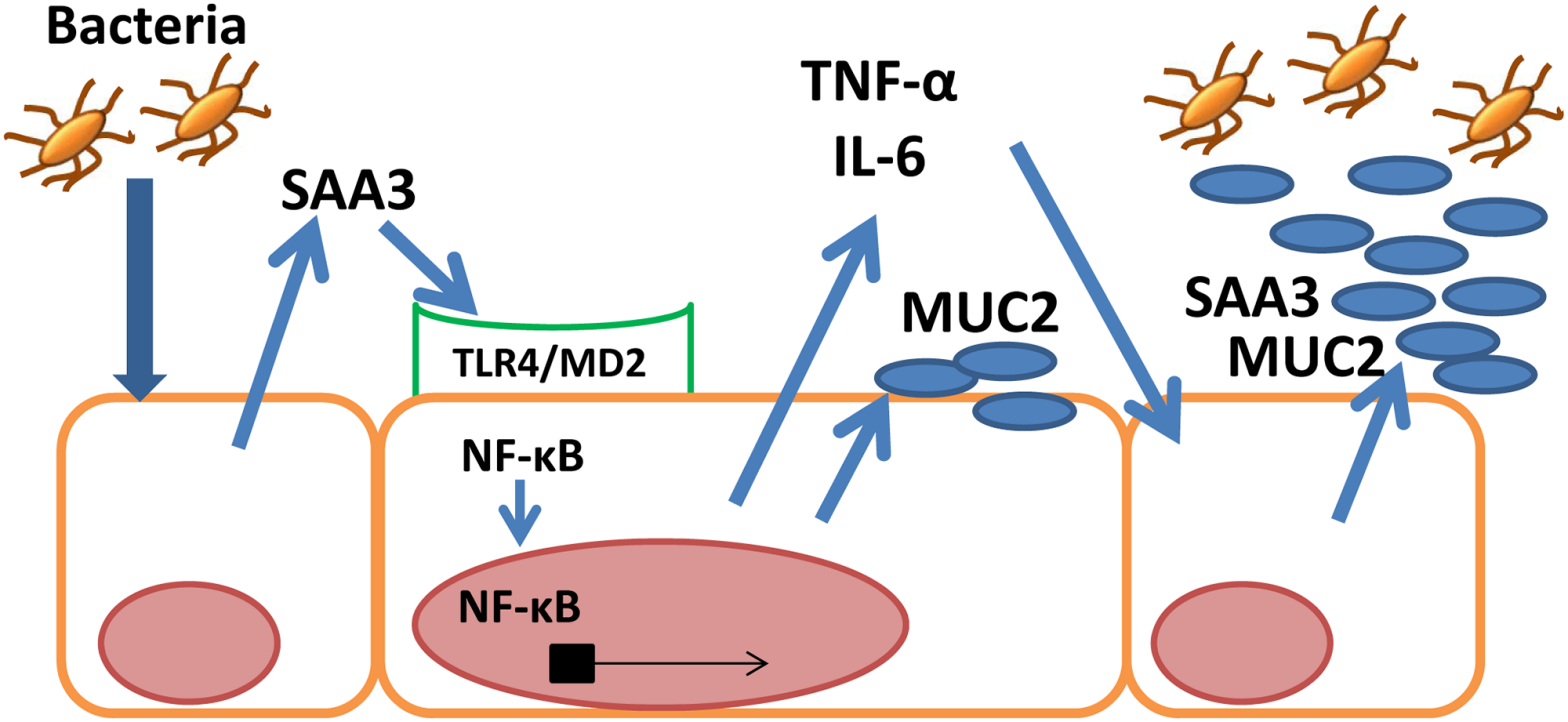
Schematic illustration of MUC2 expression by SAA3. SAA3 activates NF-κB via TLR4/MD2 and induces MUC2 expression after Gram negative bacterial infection in colonic epithelial cells. Simultaneously, SAA3 induces TNF-α and IL-6 expression, which results in further up-regulation of MUC2 by TNF-α and other mucins by IL-6, and also SAA3 expression by TNF-α and IL-6, to protect epithelial cells from bacterial infection.

In summary, this study showed that amino acids 1–36 of SAA3 induced MUC2 expression, and we propose a mechanism by which SAA3 induces MUC2 expression in CMT-93 cells after Gram negative bacterial infection (Fig 7). Interestingly, the TFLK motif in bovine mammary-associated SAA3 increases MUC3 expression in a heterologous host, human intestinal epithelial HT-29 cells [30], a finding that indicates a potential therapeutic/probiotic use of SAA3 to protect intestines from bacterial infection in humans and animals. Further investigations are needed to clarify the essential amino acid sequence of SAA3 for MUC2 expression and to understand the role of SAA in host intestinal immunity in detail.

## Supporting information

S1 Table Oligonucleotide primers used for quantitative real-time PCR. (DOCX)
