# Supplementary material for: The N-terminal region of serum amyloid A3 protein activates NF-κB and up-regulates MUC2 mucin mRNA expression in mouse colonic epithelial cells
Source: PLoS One. 2017 Jul 24;12(7):e0181796. doi: 10.1371/journal.pone.0181796 (PMC5524290; doi:10.1371/journal.pone.0181796)
Supplement: S1 Table — (DOCX) [file pone.0181796.s001.docx]

Supporting information

**S1 Table. Oligonucleotide primers used for quantitative real-time PCR**

| Primers * | Sequence (5'-3') | Reference |
| --- | --- | --- |
| MUC2 F | GCTGACGAGTGGTTGGTGAATG | Wlodarska *et al*., 2011 |
| MUC2 R | GATGAGGTGGCAGACAGGAGAC |  |
| TNF-α F | GCCTCTTCTCATTCCTGCTT | Yang *et al*., 2004 |
| TNF-α R | CACTTGGTGGTTTGCTAGGA |  |
| IL-6 F | TTCCATCCAGTTGCCTTCTT | Yang *et al*., 2004 |
| IL-6 R | ATTTCCACGATTTCCCAGAG |  |
| IκB-α F | GCAATCATCCACGAAGAGAAGC | Cadera *et al*., 2009 |
| IκB-α R | CGTTGACATCAGCACCCAAAG |  |
| REG III-γ F | TTCCTGTCCTCCATGATCAAAA | Cash *et al*., 2006 |
| REG III-γ R | CATCCACCTCTGTTGGGTTCA |  |
| α Def F | AAGAGACTAAAACTGAGGAGCAGC | Clarke *et aｌ*., 2004 |
| α Def R | GGTGATCATCAGACCCCAGCATCAGT |  |
| βDef-3 F | CCTTCTCTTTGCATTTCTCCTGG | Rahman *et al*., 2010 |
| βDef-3 R | CATTTGAGGAAAGGAACTCCACAA |  |
| βDef-4 F | TCTTCACATTTCTCCTGGTGCTGCTG | Rahman *et al*., 2010 |
| βDef-4 R | TTGCTGGTTCTTCATCTTTTTATCT |  |
| GAPDH F | TGCACCACCAACTGCTTAG | This study |
| GAPDH R | GGATGCAGGGATGATGTTC | (Accession No. GU214026) |

* MUC2, mucin 2; TNF-α, tumor necrosis factor-α; IL, interleukin; IκB-α, inhibitor of κB-α; REG III-γ, regenerating islet-derived III-γ; Def, defensin; GAPDH, glyceraldehyde-3-phosphate dehydrogenase.
